# Stability Indicating HPTLC Determination of Meloxicam

**DOI:** 10.4103/0250-474X.45406

**Published:** 2008

**Authors:** Namita Desai, Purnima Amin

**Affiliations:** Pharmaceutical Sciences and Technology Division, University Institute of Chemical Techniology (Autonomous), Univesity of Mumbai, Matunga, Mumbai-400 019, India

**Keywords:** Meloxicam, HPTLC, antiinflammatory, stability indicating

## Abstract

A simple, selective, precise and stability-indicating high-performance thin layer chromatographic method of analysis of meloxicam both as a bulk drug and in formulation has been developed. The mobile phase selected was ethyl acetate:cyclohexane:glacial acetic acid (6.5:3.5:0.02% v/v/v). The calibration curve of the drug was linear in the range of 100-500 ng. The spectrodensitometric analysis was carried out in the absorbance mode at 353 nm. The mean (±RSD) values of slope, correlation coefficient and intercept were 3183.8±0.358, 0.9996±0.0321 and 13012±7.1 respectively. The system precision and the method precision studies were carried out with RSD of 0.83 and 1.89 respectively. The limit of detection and quantitation were 30 ng and 99 ng respectively. The mean percent recovery was found to be 100.3%. The method was used to analyze meloxicam from marketed tablet formulation in the presence of commonly used excipients.

Meloxicam is a non steroidal antiinflammatory drug (NSAID) of the oxicam class, developed for the treatment of rheumatoid and osteoarthritis^1^–^3^. An electrochemical oxidation and HPLC methods for estimation of meloxicam from tablet dosage form have been reported^4^,^5^. As against HPLC, HPTLC method can be used for mobile phases greater than pH 8. Also, it can be used for the analysis of large as well as small samples. Also, over the past decade, HPTLC has been successfully used in the analysis of pharmaceuticals, plant constituents and biomacromolecules^6^,^7^. This paper describes a simple, accurate, precise and specific HPTLC method for the determination of meloxicam as bulk drug and from pharmaceutical dosage form.

Meloxicam was obtained as a gift sample from Virdev Intermediates Pvt. Ltd. Analytical grade solvents, HPLC grade solvents and reagents were purchased from Ranbaxy Fine Chemicals Ltd. (India). A stock solution of meloxicam (1 mg/ml) was prepared in chloroform. A standard solution of 100 μg/ml was used for the analysis. The samples were spotted on HPTLC aluminum plates (10×10 cm) precoated with silica gel 60 F_254_ (layer thickness 0.2 mm, E-Merck). Spotting was done using Camag Linomat IV model. The samples were spotted in the form of narrow bands of length 3 mm, 15 mm from the bottom edge, 10 mm from the margin, 5 mm apart at a constant rate of 10 s/μL using a nitrogen aspirator. The chamber was saturated for a period of 45 min and the plate was allowed a run length of 7 cm. The separation was observed under short length (254 nm) ultraviolet lamp. Densitometric analysis of the separated components was carried out using Camag TLC Scanner II (Camag, Switzerland) in the absorbance/reflectance mode at 353 nm. The slit dimensions were 4×0.3 mm and the sensitivity was kept at the auto mode. Scanning speed was 1 mm/s. Integration of the chromatogram was carried out using the Camag TLC scanner/integrator system (Perkin Elmer, USA). Appropriate volumes of standard solution (100 μg/ml) were spotted to obtain meloxicam in the concentration range of 100-1000 ng (n=3) and calibration curve was plotted.

To evaluate system precision^8^, six spots of each were applied from a single standard solution (100 μg/ml) to get concentrations of 100 and 400 ng. Method precision was carried out by applying the spots (100 and 400 ng each) from six different standard solutions (100 μg/ml). The limit of detection (LOD) was determined on the basis of signal to noise ratio. LOD was the amount of the applied sample producing a peak area that is equal to the sum of the mean blank area and three times the standard deviation. Limit of quantitation (LOQ) was the amount of the applied sample producing a peak area that is equal to the sum of the mean blank area and ten times its standard deviation. Recovery study was carried out by spiking 50% (3.7 mg) and 100% (7.5 mg) of the standard drug to the preanalysed marketed sample of Meloxicam tablets (7.5 mg). The resulting mixture was analyzed by the proposed method. Marketed meloxicam tablets were analyzed by the proposed method. The sample preparation was done by extracting the drug in chloroform, filtering and suitably diluting the solutions. The marketed formulation excipients included lactose, microcrystalline cellulose, starch and polyvinylpyrrolidone. A placebo tablet was subjected to the same extraction procedure as discussed above and spotted. The possibility of excipient interference in the analysis was studied. The degradation of meloxicam solution (1 mg/ml) was carried out using 1 M HCl and 1 N NaOH. The solutions were refluxed for 3 h, cooled and neutralized. The solutions were diluted suitably to yield a resultant solution of 100 μg/ml. An aliquot of 4 μl of these solutions was spotted on the plate and analyzed by HPTLC. The drug was also subjected to photodegradation by placing the sample in direct sunlight for 48 h, heat degradation by placing the sample at 60° for 48 h and oxidative degradation by treatment with hydrogen peroxide (30%).

HPTLC offers the advantages of automatic application under the pressure of nitrogen gas and scanning *in situ*, where the conditions can be more easily controlled. Several samples can be run simultaneously using a small quantity of mobile phase and the substances are permanently stored on the plate. A solvent system that would give dense and compact spots with appropriately and significantly different R_f_ values for meloxicam and its degraded products was desired for the quantification of meloxicam in the pharmaceuticals. The mobile phase comprising of ethyl acetate:cyclohexane:glacial acetic acid (6.5:3.5:0.02% v/v/v) gave an R_f_ value of 0.571 for meloxicam. Glacial acetic acid was used in the mobile phase to reduce the diffusion of the drug spots. The degraded products of meloxicam obtained by various treatments using base and oxidative degradation resulted in products with R_f_ values different from that of meloxicam. The R_f_ of the degraded product was found to be 0.428 (base degradation) and 0.5 (oxidative degradation), which are quite different from that of the drug (0.571). Fig. 1 shows the separation of the degradation products obtained by various methods. The developed analytical method could be considered as a stability indicating method for the analysis of meloxicam. Linearity was observed in the range of 100-500 ng. The mean values (±SD) of correlation coefficient, slope and intercept were 0.9996±0.0321, 3183.8±0.358 and 13012±7.1 respectively. Accuracy and precision studies were carried out at two levels (100 and 400 ng). The method was found to be highly accurate and precise over the linearity range as depicted in Table 1 (RSD<2). The limit of detection and limit of quantitation for meloxicam were found out to be 30 and 99 ng, respectively. The recovery of meloxicam from the formulation was determined by comparing the peak areas obtained from the formulation to which had been added meloxicam (150 or 300 ng) with the peak areas obtained from preanalysed formulation (300 ng). The results are shown in the Table 2. The mean percent recovery was found to be 100.3%. Meloxicam content from marketed tablets (7.5 mg meloxicam) was determined. A single spot at an R_f_ value at 0.571 was observed in the samples from the marketed meloxicam tablets. The drug content was found to be 100.207±1.50%. Also, a single spot at an R_f_ value at 0.571 was observed in the samples from the marketed meloxicam tablets. It could therefore be suggested that no degradation of meloxicam could be detected with this method. There was no interference from the excipients commonly present in the conventional tablets. Thus, the proposed method is simple, rapid, accurate and precise. It can be used as a stability indicating assay method for the analysis of Meloxicam as a bulk drug and from pharmaceutical dosage forms and can be extrapolated for the estimation of Meloxicam from plasma samples.

**Fig 1:**
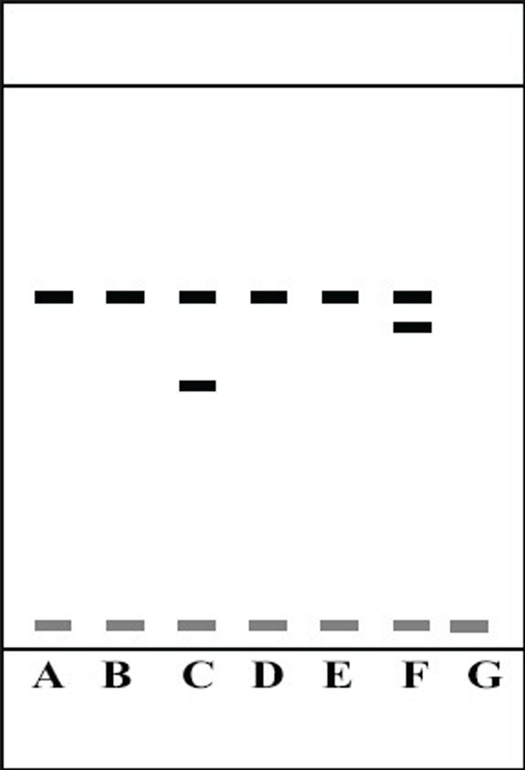
Separation of degradates of Meloxicam by HPTLC A= meloxicam standard, B= acid degradation, C= base degradation, D= heat degradation, E= photodegradation, F= oxidative degradation, G= placebo

**TABLE 1:** PRECISION OF THE ASSAY

	Area under the chromatographic peak					
	
	Method precision			System precision		
		
	100 ng		400 ng	100 ng		400 ng
	364261		1304277	368397		1301254
	348689		1285476	361387		1306894
	365689		1276578	365478		1315462
	355897		1328748	368689		1325645
	356598		1333999	368694		1325461
	365987		1325647	369658		1324689
% RSD	1.932		1.845	0.86		0.8
MEAN RSD		1.89			0.83	

RSD= Relative Standard Deviation

**TABLE 2:** RECOVERY STUDY OF MELOXICAM FROM MARKETED FORMULATION

Sample	Excess drug added to the analyte (%)	Theoretical content (ng)
M1*	0	300
	50	450
	100	600
	Mean% Recovery (+ SD) = 100.30 + 1.51	

* M1 = Marketed meloxicam tablets (7.5 mg)
